# Brightening Gold Nanoparticles: New Sensing Approach Based on Plasmon Resonance Energy Transfer

**DOI:** 10.1038/srep10142

**Published:** 2015-05-11

**Authors:** Lei Shi, Chao Jing, Zhen Gu, Yi-Tao Long

**Affiliations:** 1 Key Laboratory for Advanced Materials & Department of Chemistry, East China University of Science and Technology, Shanghai 200237, P. R. China

## Abstract

Scattering recovered plasmonic resonance energy transfer (SR-PRET) was reported by blocking the plasmon resonance energy transfer (PRET) from gold nanoparticle (GNP) to the adsorbed molecules (RdBS). Due to the selective cleavage of the Si-O bond by F^−^ ions, the quenching is switched off causing an increase in the brightness of the GNPs,detected using dark-field microscopy (DFM) were brightened. This method was successfully applied to the determination of fluoride ions in water. The SR-PRET provides a potential approach for a vitro/vivo sensing with high sensitivity and selectivity.

Localized surface plasmon resonance (LSPR) occurring in plasmonic metallic particles (Au, Ag and Cu) has attracted increasing attentions^1^,^2^,^3^,^4^. Plasmonic particles, specifically gold nanoparticles (GNPs), are widely used building blocks for the fabrication of biosensors, due to their excellent optical and chemical properties^5^,^6^,^7^,^8^. Due to the facile synthesis, modification and excellent biocompatibility, gold nanoparticles have been utilized in biomedical detection, disease diagnosis and drug delivery^9^,^10^,^11^. Their absorption and scattering could be used to monitor biomolecules and heavy metal ions^12^,^13^. For instance, reduced nicotinamide adenine dinucleotide (NADH) could reduce Cu^2+^ ions to Cu(0) to form Au@Cu core-shell nanostructures. By detecting the shift in the maximum scattering wavelength (Δλ_max_), the concentration of NADH near a single Au nanoparticle was determined^14^. Plasmon resonance Rayleigh scattering (PRRS) spectroscopy and dark-field microscopy (DFM) have both helped in determining the size, shape, composition, and the local environment of single plasmonic nanoparticles. Moreover, each individual nanoparticle can behave as an independent probe. Single nanoparticle probes provide high sensitivity and high spatial resolution.

GNPs, due to the fact that they are nontoxic and lack of photobleaching or blinking, have been used in cell imaging and in vivo biosensing. The scattering intensity of GNPs is stronger than the fluorescence of organic dyes and quantum dots (QDs)^15^. In 2007, it was reported that plasmonic resonance energy could transfer from gold nanospheres to surface modified Cytochrome c, resulting in “quantized quenching dips” in the spectra of resonant Rayleigh scattering light^16^. Similar to the donor–acceptor energy matching of Förster resonance energy transfer (FRET)^17^, the PRET only occurs when the condition of frequency matching is satisfied between the nanoparticles and the corresponding chemical or biomolecules, and single metallic nanoparticle provides high spatial resolution, the PRET-based sensors have high selectivity and high sensitivity. Therefore, more and more attention has been paid to construct nanoplasmonic probes based on PRET^18^. For example, a highly sensitive and selectively sensor was developed for the determination of Cu^2+^, with a detection limit down to 1 nM making it more sensitive than organic reporter-based methods^19^.

Despite current findings, the reported PRET based sensors are a signal-OFF type causing them to suffer from high background noise^20^. In turn, in this paper, we will report a SR-PRET phenomenon by blocking the energy transfer from the nanoparticle to the molecules. To the best of our knowledge, this is the first report on SR-PRET. We believe that our strategy will give a new sensing pathway for biological determination.

## Results

### The mechanism and the strategy of SR-PRET

**Figure 1:**
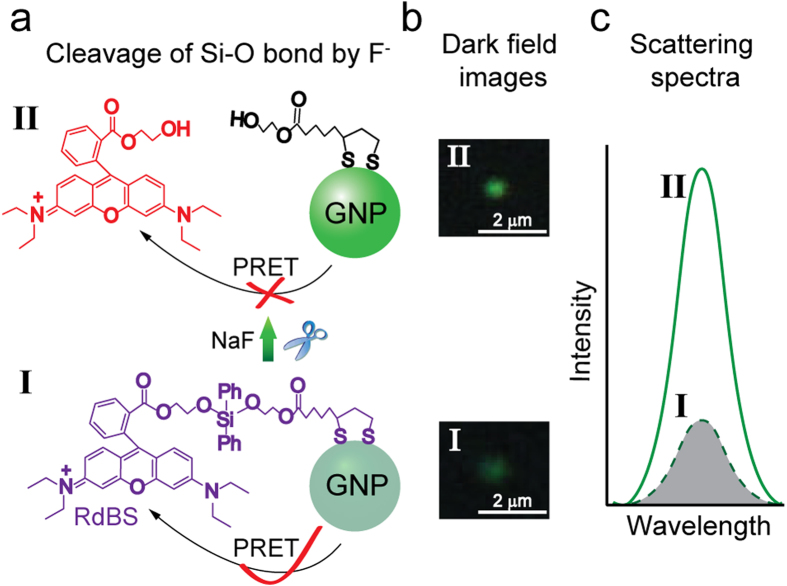
Mechanism of the process of SR-PRET. **a**) Schematic representation of the process of SR-PRET. **b**) The color images of a typical GNP before (I) and after (II) adding F^−^ ions, demonstrating the recovery of the scattering intensity. **c**) Scattering spectra intensity before (I) and after (II) the addition of F^−^ ions, the remarkable resonance enhancement on the Rayleigh scattering spectrum is clearly observed.

To study the process of SR-PRET, we designed and synthesized a compound RdBS (Fig. 1a) with an absorption peak at 565 nm in water (Figure S1). To meet the condition of PRET, 60 nm GNPs were synthesized and characterized by UV-Vis spectrometry (Figure S2). The results shown using dark-field microscopy (DFM) contain a green color and a scattering peak at ~560 nm (Figure S3a, b). The system for probing the scattering recovery process was prepared through several steps, beginning with the immobilization of 60 nm GNPs on clean glass slides. Thereafter, the slides were immersed in a mixed solution of RdBS and 1-propanethiol for 12 h, allowing the self-assembly of RdBS and 1-propanethiol onto the surface of the gold nanoparticles. Due to the electrostatic attraction between positively charged N-atoms in rhodamine molecules and negatively charged gold nanoparticles^21^, 1-propanethiol was initially allowed interact with RdBS prior to immersion. The scattering images were collected through a DFM system, and the plasmon resonance Rayleigh scattering (PRRS) spectra were recorded by a spectrometer (Figure S4).

According to the mechanism of PRET, when the plasmon resonance frequency of a gold nanoparticle is related to its specific molecular absorption band, the process of PRET takes place, resulting in wavelength quenching in the Rayleigh scattering spectrum. The absorption peak of RdBS overlaps with the scattering peak of the 60 nm gold nanoparticle, fulfilling the condition of PRET. In turn, when the RdBS molecules assembled to the 60 nm gold nanoparticles, the plasmon resonance energy of the gold nanoparticles transferred to the RdBS molecules and the quenching is observed in the Rayleigh scattering spectra (Figure S3c, d). The RdBS contains a Si-O bond which is introduced as a reaction site for F^−^ ions. Because of the high affinity of F^−^ for silicon, the reaction of F^−^ with RdBS would trigger the cleavage of Si-O bond to release the rhodamine group (Fig. 1a), brighten the GNPs (Fig. 1b) and recover the scattering (Fig. 1c).

**Figure 2:**
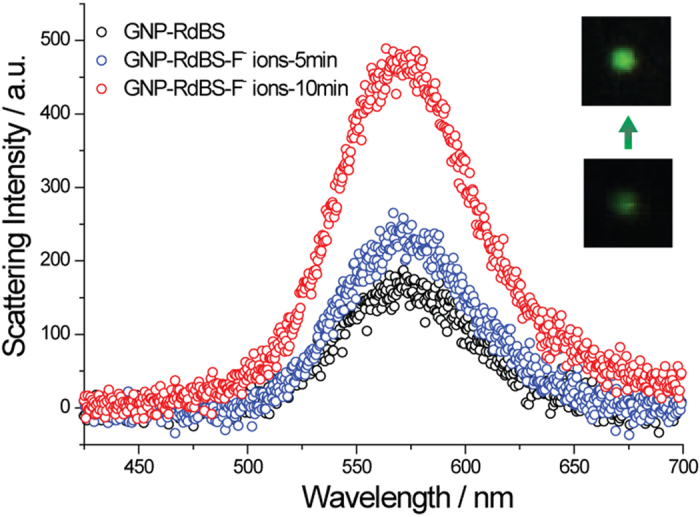
Plasmon resonance Rayleigh scattering spectra of RdBS modified gold nanoparticles after exposure to 1 μM NaF. The inset shows the color images of a typical gold nanoparticle.

To investigate the sensitivity of the SR-PRET sensing system, changes in the plasmon scattering spectra were recorded upon adding a solution of NaF. After 10 minutes, the Rayleigh scattering peak (~560 nm) of a single gold nanoparticle exhibits an increase in intensity without noticeable spectra shift and the particle is brightened (Fig. 2). The TEM images show that the size of the gold nanoparticles were not changed before and after the addition of F^−^ ions (Figure S5), indicating that such intense quenching is not caused by the dimension change of the gold nanoparticle. Therefore, we can conclude that the recovery of the scattering intensity was induced by the addition of F^−^ ions which trigger the cleavage of the Si-O bond^22^.

The interaction between RdBS and F^−^ ions was confirmed by UV-Vis spectroscopy in an aqueous solution. In the absence of F^−^, the maximum absorption wavelength of RdBS is about 565 nm. When 10 equiv. of NaF was added to the aqueous solution of RdBS (1.0 × 10^−5^ M), the absorption spectrum of RdBS shifted from ~565 nm to ~553 nm and the color of the solution was changed from purple to red (inset of Figure S1). To confirm the mechanism illustrated in Fig. 1, the UV-Vis spectrum of compound **2** was investigated (Figure S1). As expected, compound 2 has an absorption band at around 553 nm which is indicated in the absorption peak of the reaction product, confirming the formation of compound 2 after the addition of F^−^. The mechanism was also investigated by TLC analysis, Mass spectroscopy and IR spectroscopy. The reaction of RdBS with F^−^ under the same conditions was monitored by thin layer chromatography (TLC) analysis (Figure S6). It showed that the reaction was very clean and the reaction product analyzed by MS corresponded to compound 2 (Figure S7). The IR spectra before and after the addition of F^−^ were added (see Figure S15, S16). We can see that after the addition of fluoride ions, the infrared absorption characteristic peaks at 1129 cm^−1^ which is assigned to the Si-O bond disappeared, and a small peak at ~3600 cm^−1^ appeared which can be assigned to the free –OH. Taken together, we can confirm that the SR-PRET switch was triggered by cleavage of the rhodamine units from the nanoparticle by F^−^ ions.

**Figure 3:**
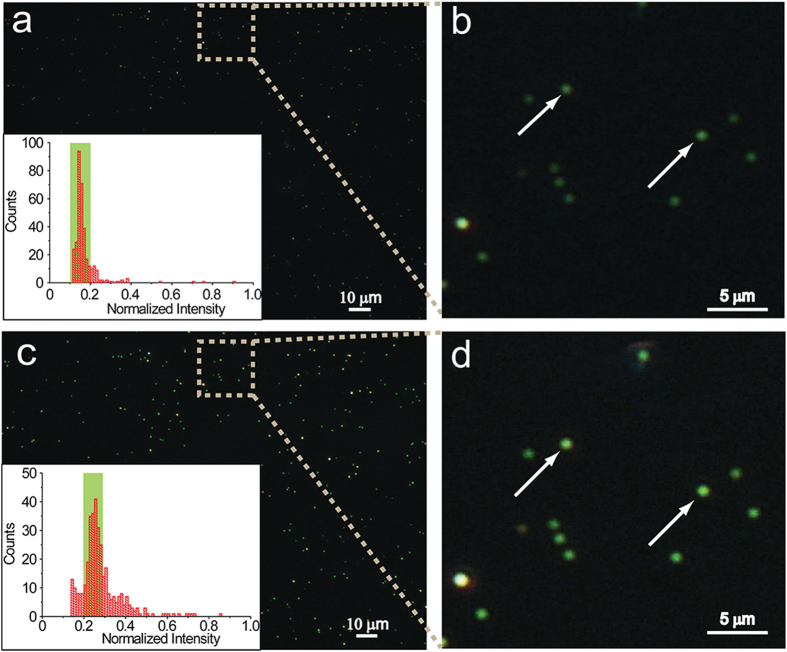
Dark-field images and scattering intensity calculation before and after addition of F^−^ ions. **a**) Dark-field image of the RdBS-functionalized 60 nm GNPs before addition of 1.0 μM NaF. **b**) The detail view of DFM image in (**a**). **c**) Calculated intensity of the RdBS-functionalized 60 nm GNPs before addition of 1.0 μM NaF. **d**) Dark-field image of the RdBS-functionalized 60 nm GNPs after the addition of 1.0 μM NaF. **e**) The detail view of DFM image in (**d**). **f**) Calculated intensity of the RdBS-functionalized 60 nm GNPs after the addition of 1.0 μM NaF.

To confirm that the SR-PRET is not just a random occurrence, a large field of the gold nanoparticles before and after addition of F^−^ ions was done. As shown in Fig. 3, the gold nanoparticles had low brightness before the addition of NaF, however, these particles were brightened after the addition of F^−^ ions. Moreover, the scattering intensity of all of the GNPs in the images were calculated from the information obtained from the brightness by Matlab program (Supplementary Information). We could see from the statistical graphs that the scattering intensity of the majority of nanoparticles was enhanced. This calculated statistic data of 350 nanoparticles confirms that the method is highly reliable.

### Sensitive and Selective determination of F^−^

Having established the SR-PRET sensing model, we tried to utilize it to selectively detect the F^−^ ions in water. The selective cleavage of Si-O bond by F^−^ ions suggests that the sensor might behave as an optical sensor for the determination of F^−^ ions. Fluoride is of particular interest owing to its essential role in the environment, medical field and as an important component in various organic syntheses^22^,^23^,^24^. Low levels of F^−^ have been shown to be effective for dental care^25^; however, excess F^−^ can lead to fluorosis^26^. As a result, the detection and selectively monitoring of F^−^ anions is of current interest^27^,^28^,^29^,^30^. In this paper, we applied the SR-PRET in the determination of F^−^ ions in an aqueous solution.

**Figure 4:**
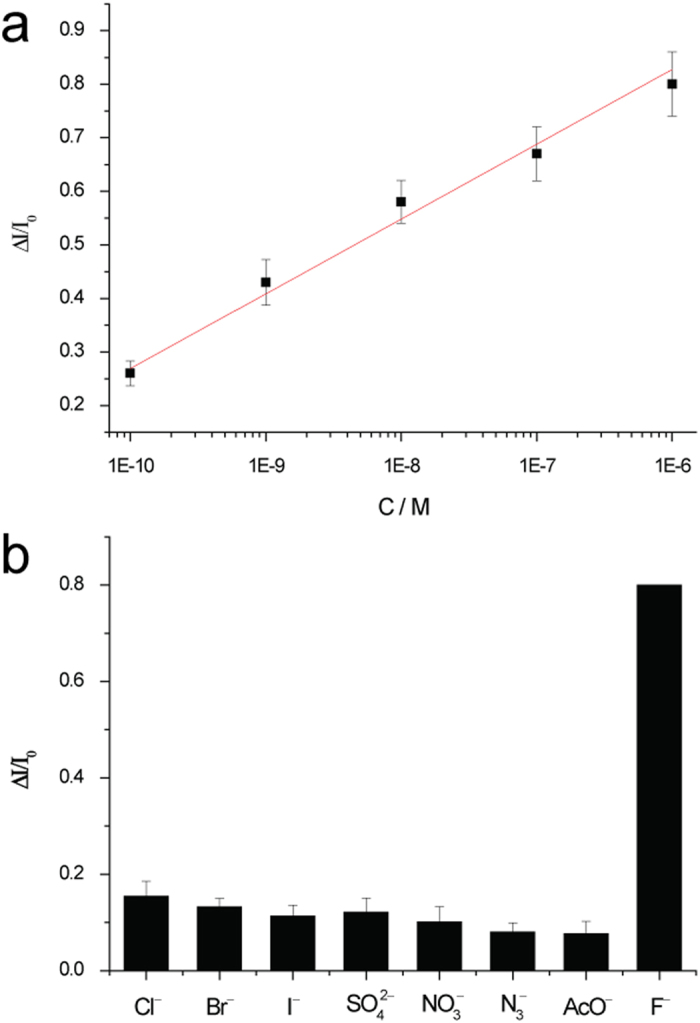
Sensitive and selective determination of F^−^ ions. **a**) Equilibrium differential scattering intensity change, ΔI/I_0_ as a function of F^−^ concentration. The red line is a logarithmic fitted to the F^−^ concentration data points. **b**) The scattering changes in response of the functionalized gold nanoplasmonic probe to various anions.

To investigate the sensitivity, different concentrations of F^−^ from a single stock solution were tested. The scattering intensity of single RdBS-modified GNP increases along with the increasing F^−^ concentrations. As shown in Fig. 4a, a linear relation between the ΔI/I_0_ and the F^−^ concentrations is observed. The regression coefficient in the equilibrium curve is 0.995 for concentrations in the 10^−10^ M to 10^−6^ M range. It is remarkable that the scattering intensity increased about 26% after the addition of 0.1 nM F^−^ to the probing system. The limit of detection, calculated as three times the blank standard deviation, was 0.072 nM.

The behavior of the SR-PRET was also tested by treatment of the RdBS-modified GNP with 1.0 μM various anions as sodium salts in water, such as Cl^−^, Br^−^, I^−^, SO_4_^2−^, NO_3_^−^, N_3_^−^ and AcO^−^. As shown in Fig. 4b SR-PRET was not found in the absence of F^−^ ions, and the increase of the scattering intensity and the imaging signal in DFM was observed upon the addition of the F^−^ ions. These results confirm that such an increase in the scattering intensity was not caused by the dissociation of RdBS from the surface of nanoparticles, but from the fluoride-induced Si-O bond cleavage. Subsequently, the PRET from gold nanoparticle to the molecules is blocked, and the Rayleigh scattering recovery could be observed. These results indicate that the SR-PRET system is a suitable sensor for selectively recognizeing fluoride ions.

### Sensing behavior of SR-PRET in cells

**Figure 5:**
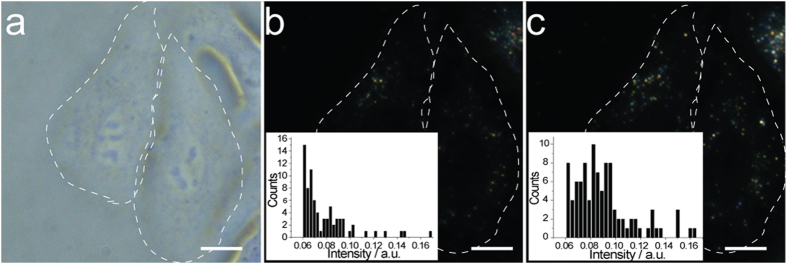
F^−^ ions determination in living cells. **a**) Bright-field images of HeLa cell after 24 h incubation with RdBS modified gold nanoparticles. **b**) DFM images of corresponding HeLa cell in (**a**). **c**) DFM images of HeLa cell in (**a**) with incubation in TBS containing 10.0 μΜ NaF for 2 h. The scale bar is 20 μm.

The determination of F^−^ with our sensing system in living Hela cells was also investigated (Fig. 5). As expected, the DFM images show that the scattering light intensity of RdBS modified gold nanoparticles was enhanced with the addition of F^−^ in a single cell. These results suggest that the sensing system could behave as an in vivo sensor.

## Conclusion

In conclusion, we reported a scattering recovery based plasmonic resonance energy transfer (SR-PRET) approach at single nanoparticle level. The fluoride-induced cleavage of Si-O bond results in the release of a rhodamine group which blocks the energy transfer from the gold nanoparticles to the molecules and thereafter the scattering light can be recovered. The method was applied to detect F^−^ with high sensitivity and selectivity in an aqueous solution confirmed via cell imaging. We believe that the SR-PRET approach can provide a new pathway to construct sensors of great sensitivity and selectivity for biological and environmental applications.

## Methods

### Materials

All the chemicals were of analytical grade and used as received. All solutions were prepared with ultrapure water (18 MΩcm) from a Millipore system. ^1^H NMR and ^13^C NMR were acquired in CDCl_3_ on BRUKER AVANCE 500 spectrometer using TMS as an internal standard. HRMS were obtained on HP5989 mass spectrometer. The dark-field spectrum measurements were carried out on an inverted microscope (eclipse Ti-U, Nikon, Japan) equipped with a dark field condenser (0.8 < NA < 0.95), a 100 W halogen lamp, a true-color digital camera (Nikon DS-fi), a monochromator (Acton SP2300i) equipped with a spectrograph CCD (CASCADE 512B, Roper Scientific) and a grating (grating density: 300 L/mm; blazed wavelength: 500 nm). The true-color scattering images of gold nanoparticles were taken using a 40X objective lens (NA = 0.8). The scattering spectra from the individual nanoparticles were corrected by subtracting the background spectra taken from the adjacent regions without the GNPs and dividing it with the calibrated response curve of the entire optical system. The spectra were integrated for 10 seconds.

### Cell culture

HeLa cells were cultured in Dulbecco’s modified Eagle’s medium (DMEM), supplemented with 10% heat-inactivated fetal bovine serum (FBS) and antibiotics (100 mg/mL streptomycin and 100 U/mL penicillin) at 37 °C in the humidified atmosphere with 5% CO_2_. The cells were seeded in 6 cm dishes at a density of 2 × 10^4^ cells/dish and grew overnight. Then cells were incubated with fresh media containing 0.15 nM GNPs (v/v, 8:1) for 24 hours. Then cells were rinsed by Tris-buffered saline (TBS, 10 mM, pH = 7.3, 0.15 M NaCl). The SR-PRET in living cell was performed in a culture medium containing 1 μM NaF after recording the original spectra of GNPs in cell.

### Synthesis and characterization of 60 nm gold nanoparticles and compound RdBS

The characterization of compounds and nanoparticles was performed as described in the Supplementary Information.

## Additional Information

**How to cite this article**: Shi, L. *et al.* Brightening Gold Nanoparticles: New Sensing Approach Based on Plasmon Resonance Energy Transfer. *Sci. Rep.* **5**, 10142; doi: 10.1038/srep10142 (2015).

## Supplementary Material

Supporting Information
