# Supplementary material for: Brightening Gold Nanoparticles: New Sensing Approach Based on Plasmon Resonance Energy Transfer
Source: Sci Rep. 2015 May 11;5:10142. doi: 10.1038/srep10142 (PMC4650782; doi:10.1038/srep10142)
Supplement: Supporting Information [file srep10142-s1.pdf]

Supplementary Information

Brightening Gold Nanoparticles: New Sensing Approach Based on Plasmon Resonance Energy Transfer

Lei Shi, Chao Jing, Zhen Gu, Yi-Tao Long*

Contents

Synthesis and characterization of 60 nm gold nanoparticles

Figure S1

Normalized absorption spectra of RdBS and RdBS+F^-.

Figure S2

Characterization of gold nanoseeds and 60 nm GNPs by UV-Vis and SEM.

Figure S3

Dark-field images and scattering spectra of GNPs before and after modification of RdBS.

Figure S4

Detailed experimental configuration of a dark-field microscopy system.

Figure S5

TEM images of RdBS modified GNPs (a) and after (b) the addition of fluoride ions.

Figure S6

TLC identification test before and after the reaction between RdBS and F^-.

Figure S7

MS spectrum of the product after the reaction of RdBS with F^-.

Synthesis of compound RdBS

Figure S8

^{1}H NMR of compound 1

Figure S9

IR spectrum of compound 1.

Figure S10

^{1}H NMR of compound 2

Figure S11

IR spectrum of compound 2.

Figure S12

^{1}HNMR of RdBS.

Figure S13

^{13}C NMR of RdBS.

Figure S14

HRMS of RdBS.

Figure S15

IR spectrum of RdBS.

Figure S16

IR spectrum of the product after the reaction of RdBS with F^-.

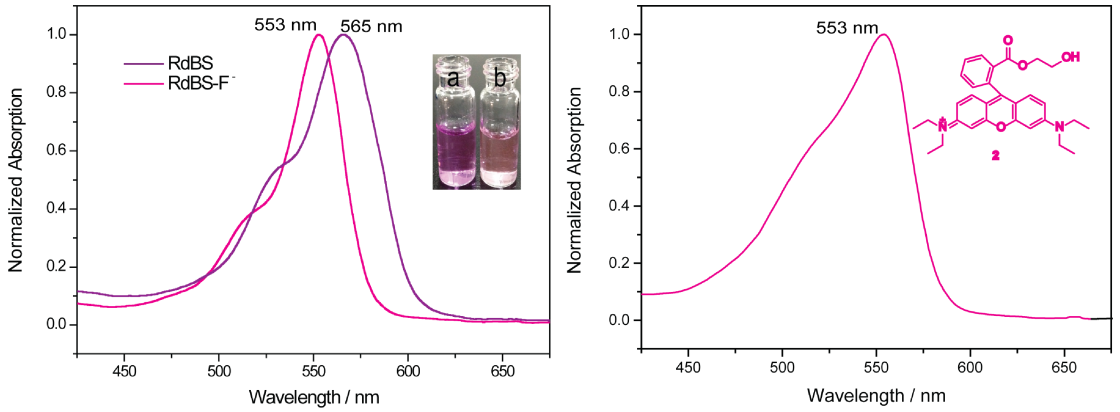

Figure S1. a) Normalized absorption spectra of RdBS and RdBS+F^-. Absorption spectra of RdBS (1.0×10^{-5} M) before (red line) and after (black line) the addition of NaF (10.0 equiv.) in water. The inset is the photograph of (a) RdBS solution, (b) RdBS +NaF. b) Absorption spectrum of compound 2 in water.

Synthesis and characterization of 60 nm gold nanoparticles[1]

Seed gold nanoparticles were prepared by the citrate-mediated reduction of $HAuCl_4$. 50 mL of 0.01 wt% $HAuCl_4$ was heated to reflux with vigorous stirring and then 5 mL sodium citrate (38.8 mM) was added quickly to the solution. The mixed solution was continued to heat for 15 min, stopped heating and kept stirring for an additional 15 min. The resulting solution of colloidal particles was filtered and characterized by an absorption maximum at 521 nm using an Ocean optical USB 2000+ UV-Vis spectrometer. These particles were then used as seed particles for the synthesis of 60 nm gold particles. To 25 mL of water, 1 mL of preformed seed gold particles and 100 μL of 0.2 M $NH_2OH{\cdot}HCl$ was mixed. The mixture was stirred vigorously at room temperature and 3.0 mL of 0.1 wt% $HAuCl_4{\cdot}3H_2O$ was added drop-wise. Diameters were characterized by UV-Vis and SEM.

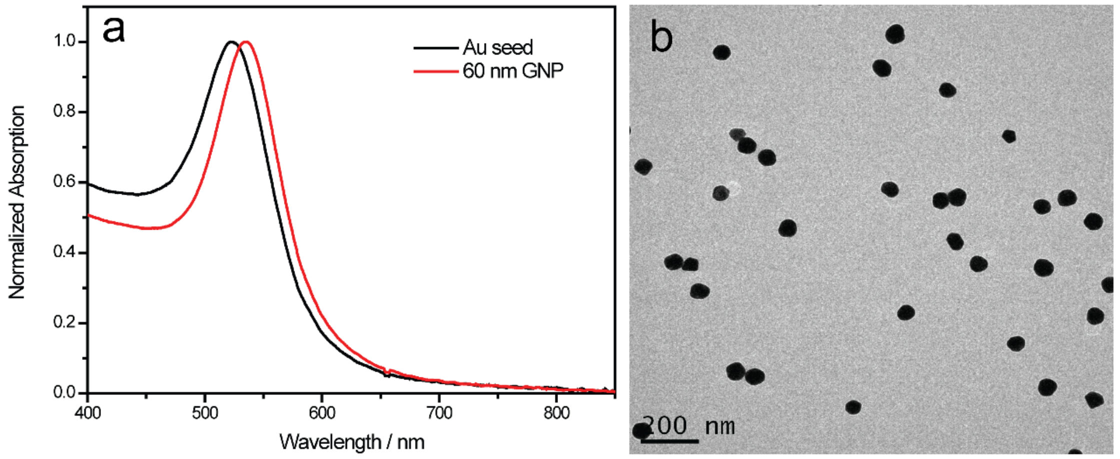

Figure S2. Characterization of gold nanoseeds and 60 nm GNPs by UV-Vis and SEM. a) UV-Vis spectrum of Au seeds and 60 nm gold nanoparticles; **b**) TEM image of 60 nm gold nanoparticles.

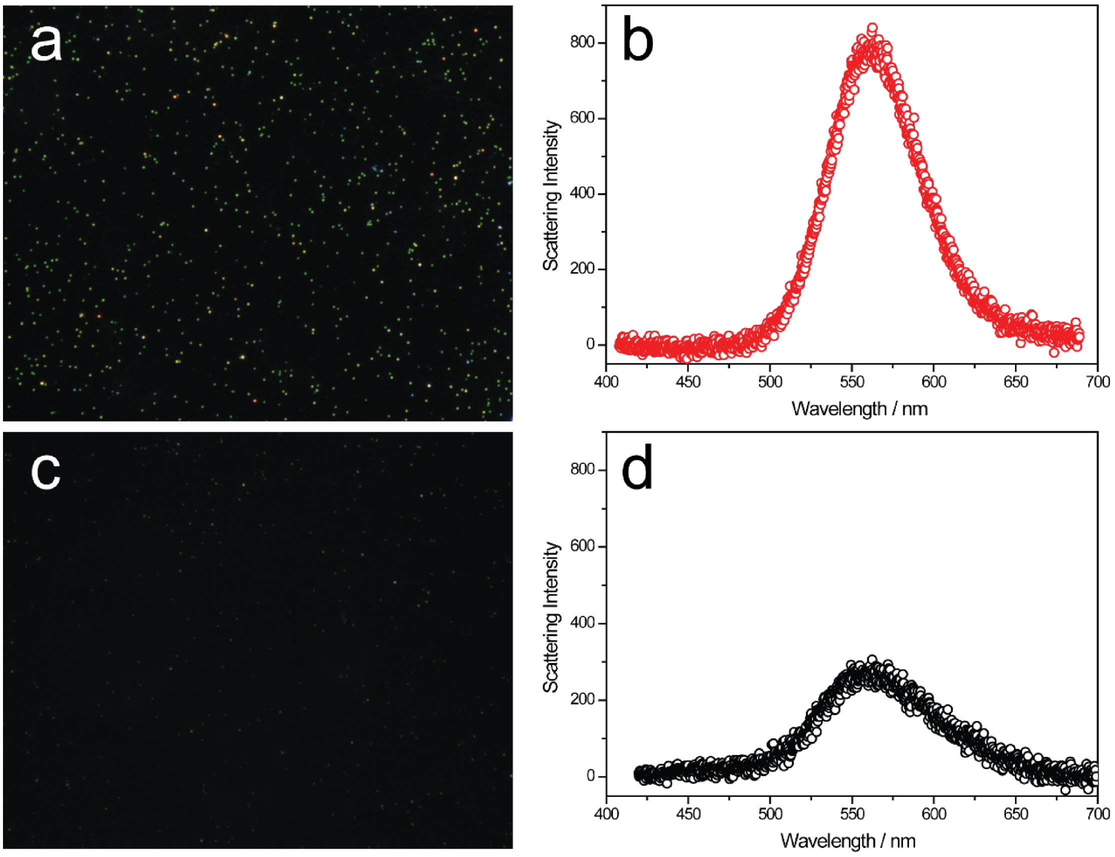

Figure S3. Dark-field images and scattering spectra of GNPs before and after modification of RdBS. a) The dark-field image of 60 nm gold nanoparticles. **b**) The scattering spectrum of a typical gold nanoparticle in (**a**); **c**) The dark-field image of RdBS modified 60 nm gold nanoparticles; **d**) The scattering spectrum of a single gold nanoparticle in (**c**).

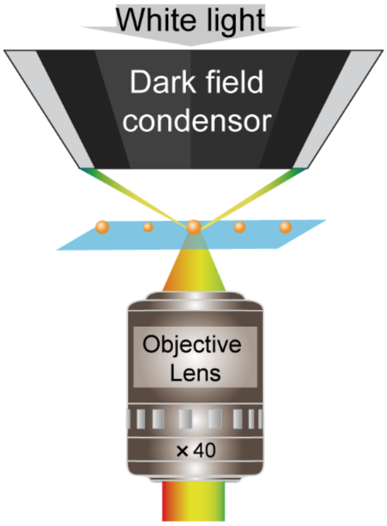

Figure S4. Detailed experimental configuration of a dark-field microscopy system. The collected scattering light is imaged by a true color charge-coupled device (CCD) camera and analyzed by a spectrometer.

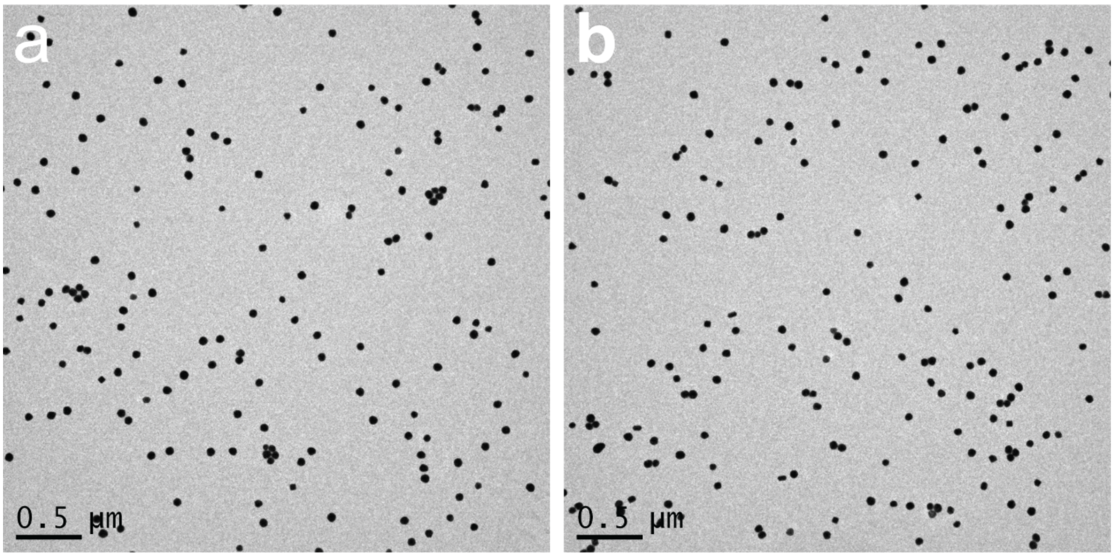

Figure S5. TEM images of RdBS modified GNPs (a) and after (b) the addition of fluoride ions.

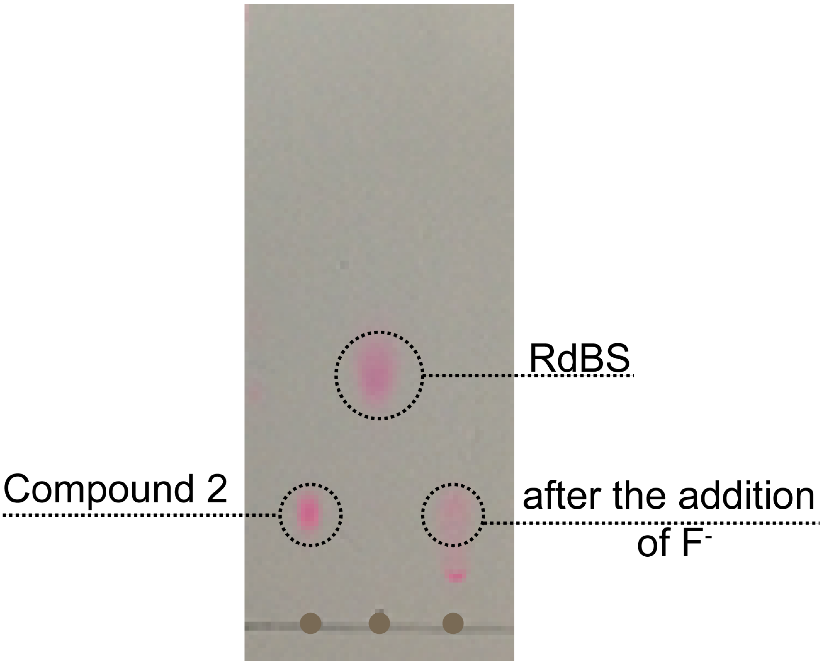

Figure S6. TLC identification test before and after the reaction between RdBS and F^-.

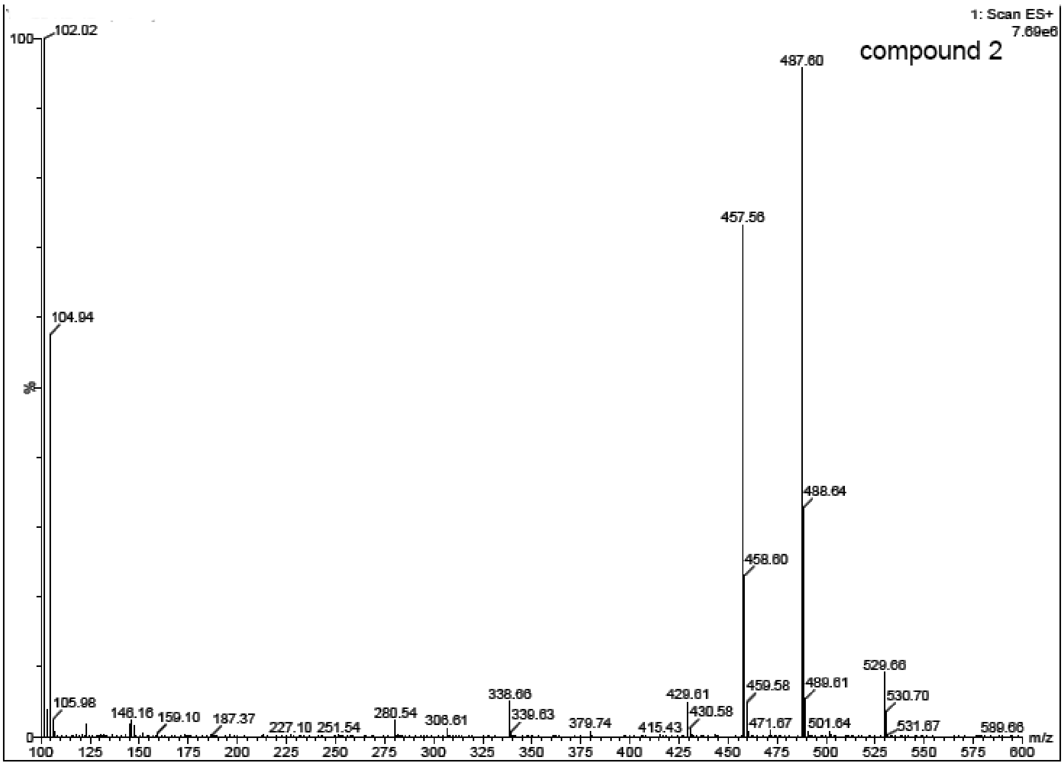

Figure S7. MS spectrum of the product after the reaction of RdBS with F^-.

Synthesis of compound RdBS

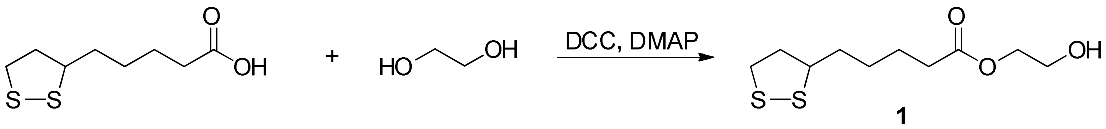

1

Glycol (3.0g, 48.5 mmol), DCC (1.03 g, 5 mmol) and DMAP (0.6 g, 5 mmol) were dissolved in 20 mL dry DCM, a solution of lipoic acid (1.0 g, 48.5 mmol) in 10 mL dry DCM was added dropwise at 0 ºC. Then the mixture was stirred at room temperature for 5 h before filtered. The filtrate was washed with brine, $NaHCO_3$ and concentrated in vacuum. The residue was purified by silica gel column chromatography to obtain the pure product as a yellow oil **1** (2.1 g). ^1H NMR (400 MHz, $CDCl_3$) δ 4.24 – 4.20 (m, 2H), 3.83 (dd, J = 9.3, 5.6 Hz, 2H), 3.58 (dq, J = 12.7, 6.4 Hz, 1H), 3.24 – 3.05 (m, 2H), 2.53 – 2.42 (m, 1H), 2.42 – 2.33 (m, 2H), 2.06 (t, J = 5.7 Hz, 1H), 1.92 (dq, J = 13.8, 6.9 Hz, 1H), 1.78 – 1.60 (m, 3H), 1.57 – 1.38 (m, 2H).

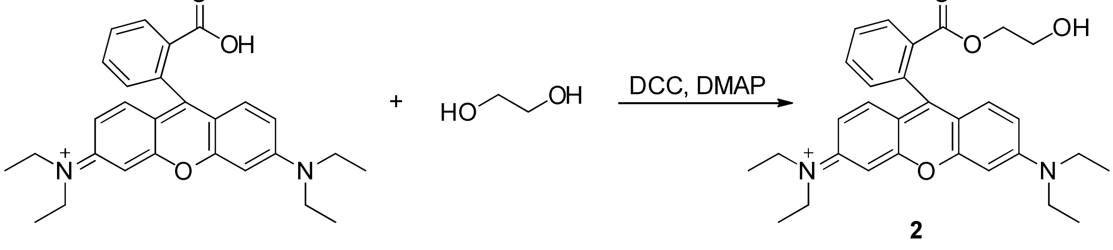

2

The synthetic procedure of 2 was the same as that of 1. ^1H NMR (400 MHz, $CDCl_3$) δ

8.48 – 8.39 (m, 1H), 7.80 – 7.69 (m, 2H), 7.26 (dd, J = 6.9, 1.5 Hz, 1H), 7.11 (d, J = 9.5 Hz, 2H), 6.88 (dd, J = 9.5, 2.2 Hz, 2H), 6.82 (d, J = 2.1 Hz, 2H), 4.18 – 4.10 (m, 2H), 3.63 (dd, J = 14.4, 7.1 Hz, 10H), 2.33 – 2.18 (m, 4H), 1.33 (t, J = 7.1 Hz, 12H).

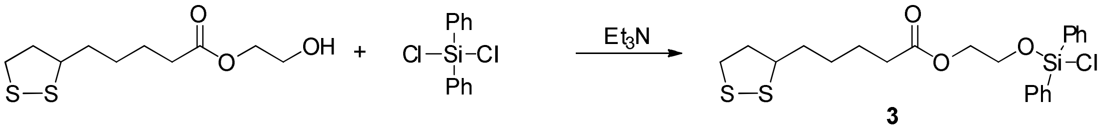

Diphenyldichlorosilane (2.28 mL, 1.1 mmol), Et_3N (2.09 mL) was dissolved in50 mL dry DCM, then a solution of 1 (2.75 g, 1.1mmol) in 20 mL dry DCM was added dropwise at 0 ºC under the protection of argon. The solution was stirred at room temperature for 6 h, then refluxed for 36 h. The solvent was concentrated in vacuum and redissolved in 1:1 ether-hexane 10 mL. The solution was suction filtered and reconcentrated. The crude product 3 was used without further purification for the next step.

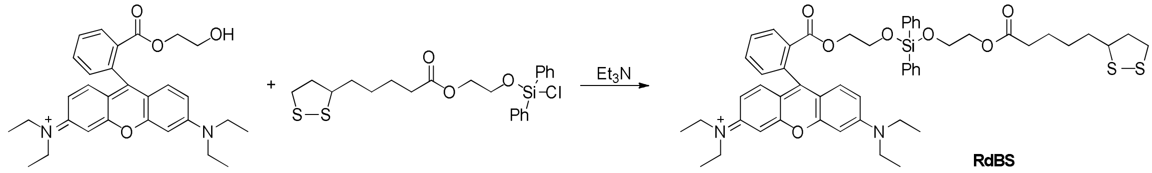

Compound 2 (0.5 g, 1.0 mmol) and Et_3N (2 mL) were dissolved in 5 mL dry DCM, a solution of crude compound 3 (0.5 g) in 5 mL dry DCM was added dropwise at 0 ºC. The mixture was stirred at room temperature for 3 h, and then quenched with saturated $NaHCO_3$. The solution was wash with H_2O, brine. The residue was purified by silica gel column chromatography to obtain the pure product **RdBS** 0.3g. ^1H NMR (400 MHz, $CDCl_3$) δ 8.17 (d, J = 7.9 Hz, 1H), 7.84 (t, J = 7.5 Hz, 1H), 7.68 (t, J = 7.7 Hz, 1H), 7.47 – 7.35 (m, 5H), 7.34 – 7.28 (m, 5H), 7.23 – 7.16 (m, 1H), 6.96 (t, J = 8.5 Hz, 2H), 6.85 – 6.69 (m, 4H), 4.12 – 4.02 (m, 2H), 3.87 – 3.78 (m, 2H), 3.74 (s, 2H), 3.60 (m, 8H), 3.12 (m, 3H), 2.45 – 2.34 (m, 1H), 2.19 – 2.00 (m, 4H), 1.86 (m, 6H), 1.58 (m, 6H), 1.36 – 1.17 (m, 12H). ^{13}C NMR (101 MHz, $CDCl_3$) δ 173.87, 173.39, 157.74, 155.50, 134.88, 134.73, 133.31 – 133.20, 132.03, 131.70131.29, 130.73, 130.43, 129.61 – 129.41, 127.92, 113.85, 96.35, 66.08, 65.96 – 65.34, 65.14, 61.22, 56.35, 51.03, 46.12, 40.24, 38.50, 34.57, 34.23 – 33.72, 31.94, 29.71, 28.75, 24.60, 22.70, 14.14, 12.66. HRMS m/zcalcd for (M+) $C_{52}H_{61}N_2O_7S_2Si$, 917.3684; found 917.3688

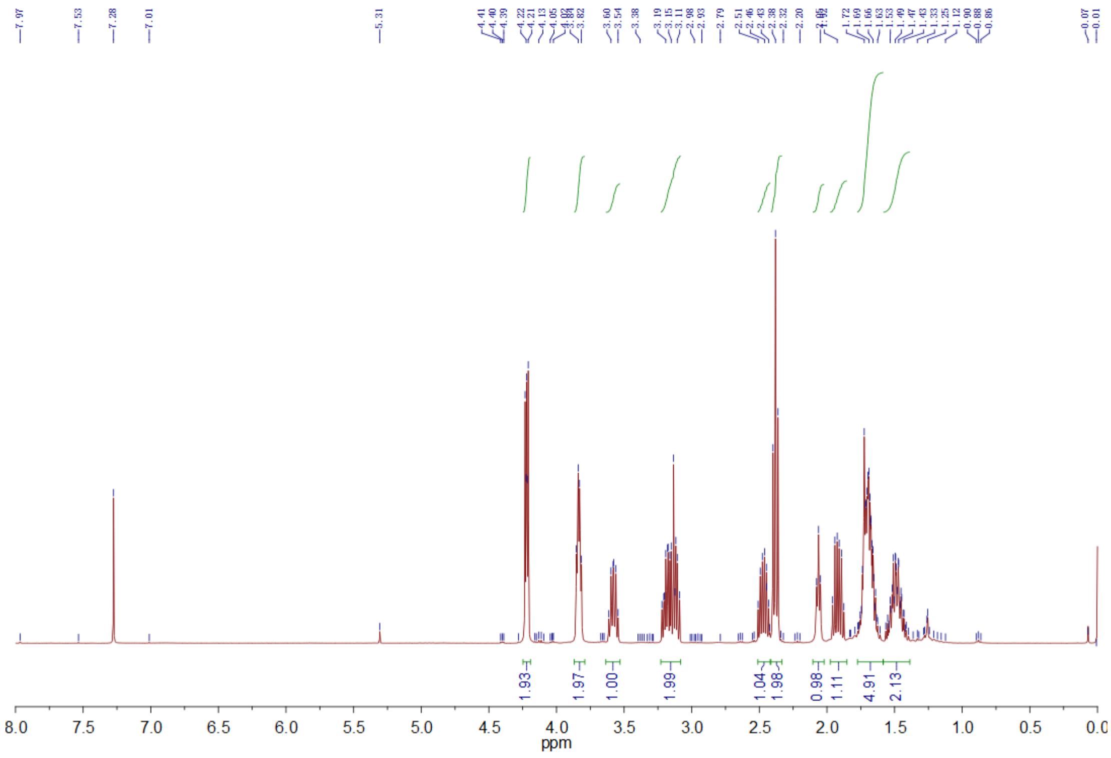

Figure S8. ^{1}H NMR of compound 1

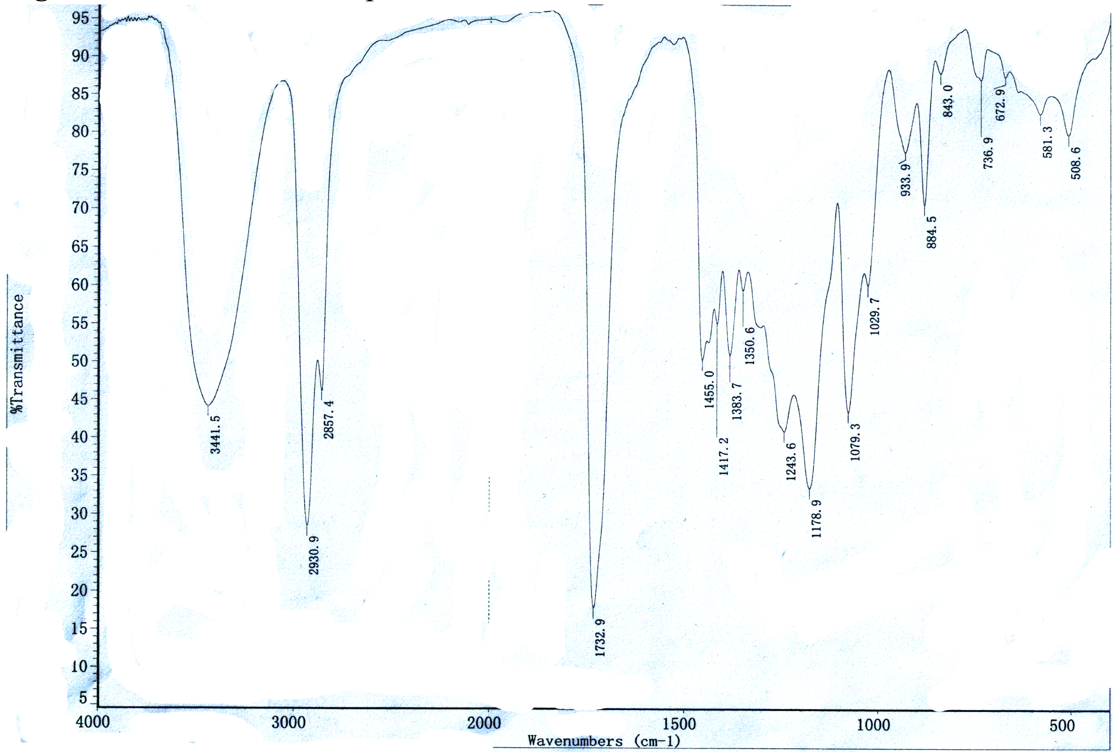

Figure S9. IR spectrum of compound 1.

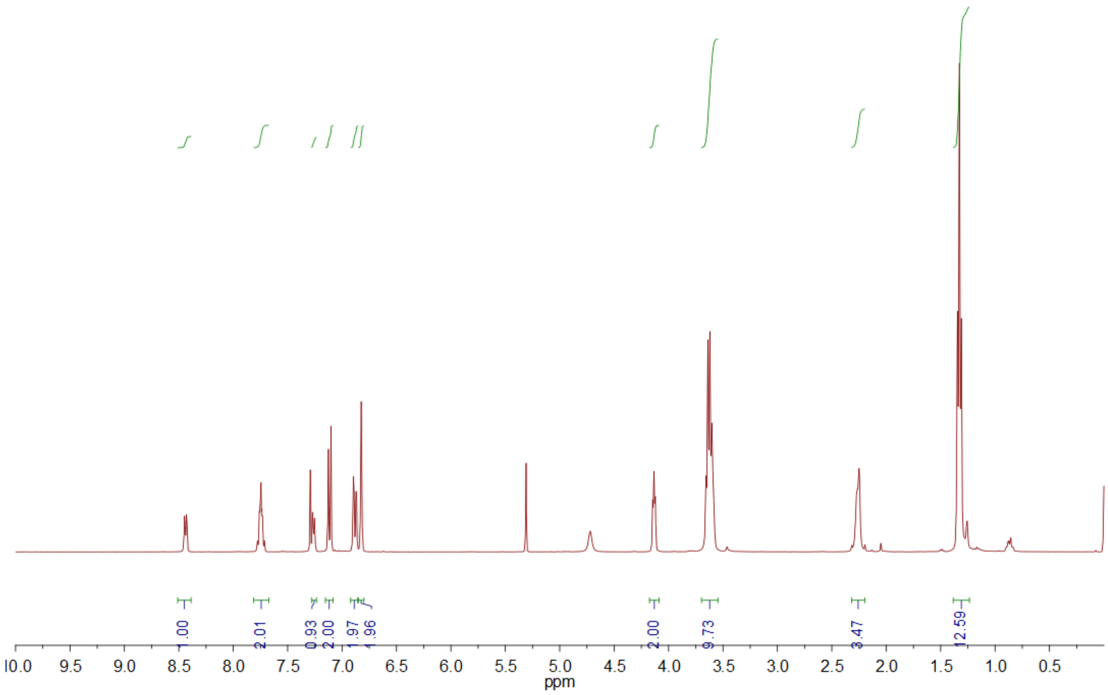

Figure S10. ^{1}H NMR of compound 2

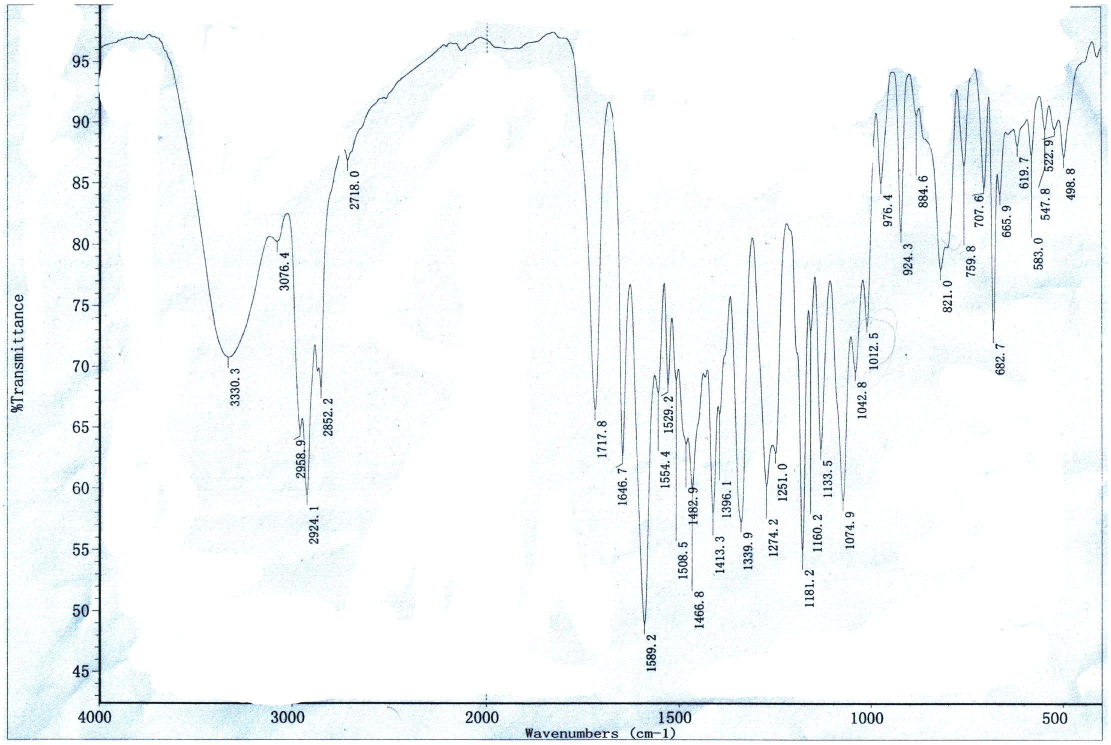

Figure S11. IR spectrum of compound 2.

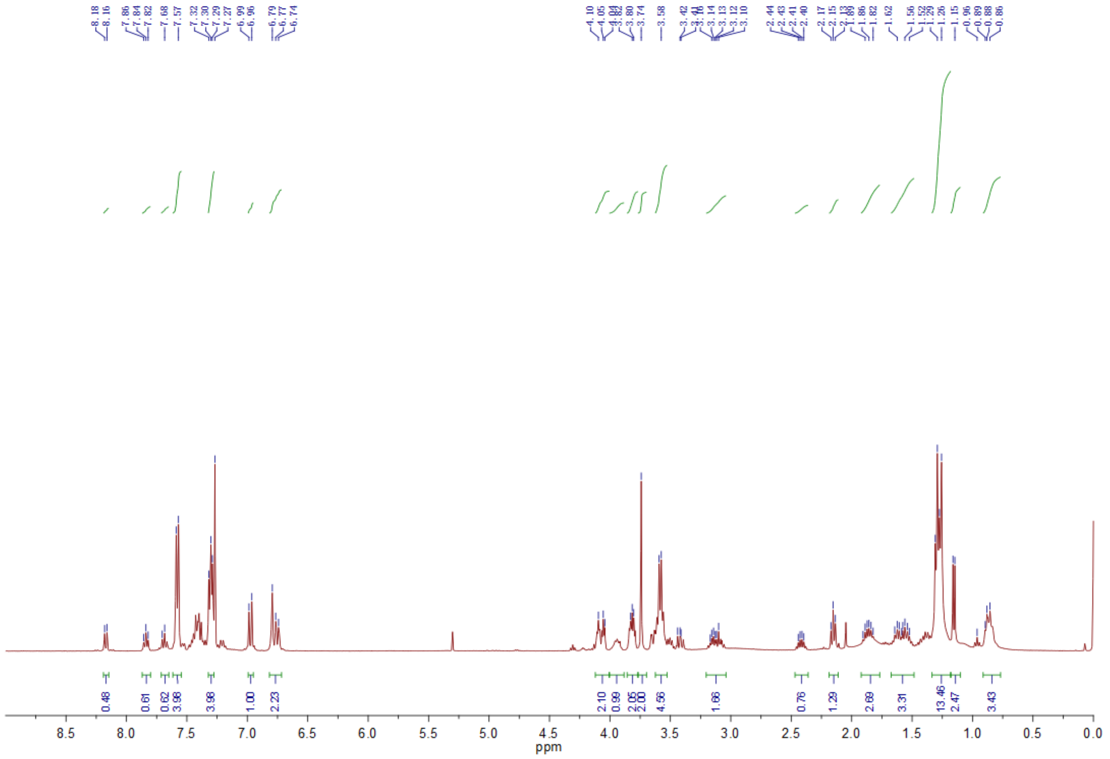

Figure S12. ^{1}HNMR of RdBS.

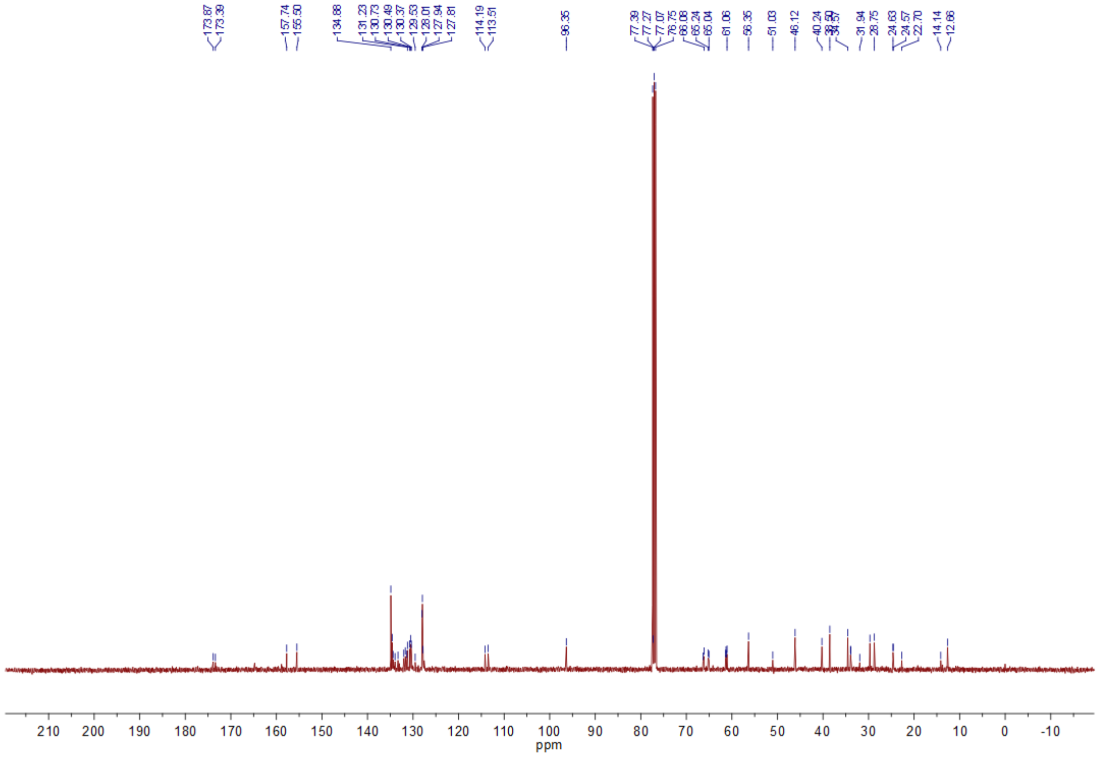

Figure S13. ^{13}C NMR of RdBS.

Elemental Composition Report

Single Mass Analysis

Tolerance = 30.0 mDa / DBE: min = -1.5, max = 100.0
Element prediction: Off
Number of isotope peaks used for i-FIT = 2

Monoisotopic Mass, Even Electron Ions
708 formula(e) evaluated with 3 results within limits (up to 1 best isotopic matches for each mass)
Elements Used:
C: 0-52 H: 0-61 N: 0-2 O: 0-7 28Si: 0-1 29Si: 0-1 30Si: 0-1 S: 0-3

LONG-YT ECUST institute of Fine Chem 06-Jun-2013 13:11:18

LYT-SL-09 21 (0.223) Cm (21:29) 1: TOF MS ES+ 1.02e+002

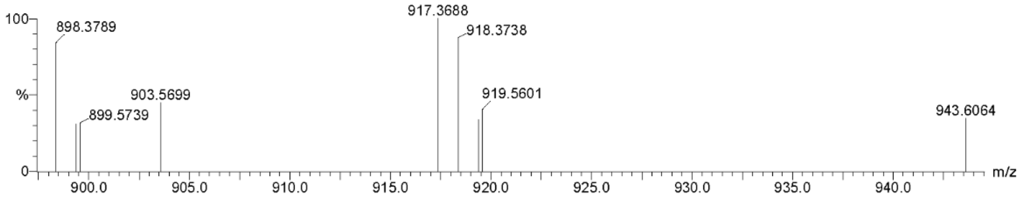

Minimum:				-1.5			
Maximum:		30.0	50.0	100.0			
Mass	Calc. Mass	mDa	PPM	DBE	i-FIT	i-FIT (Norm)	Formula
917.3688	917.3689	-0.1	-0.1	24.5	9.3	0.0	C52 H61 N2 O7 28Si S2

Figure S14. HRMS of RdBS.

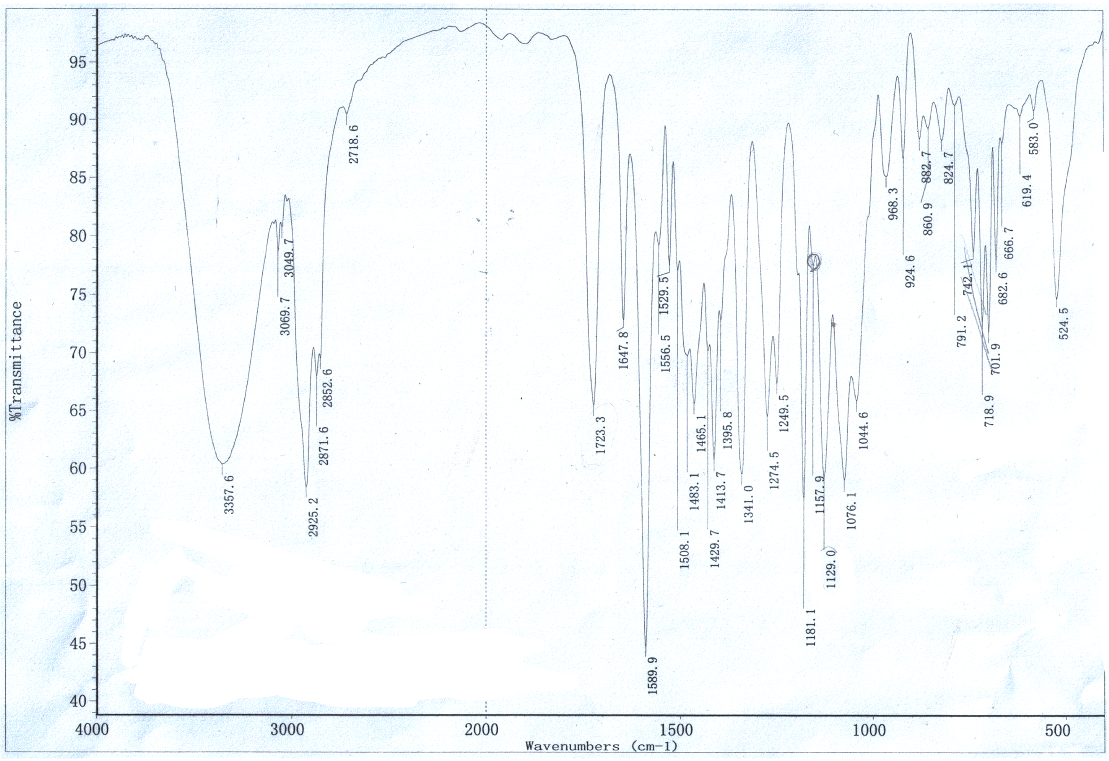

Figure S15. IR spectrum of RdBS.

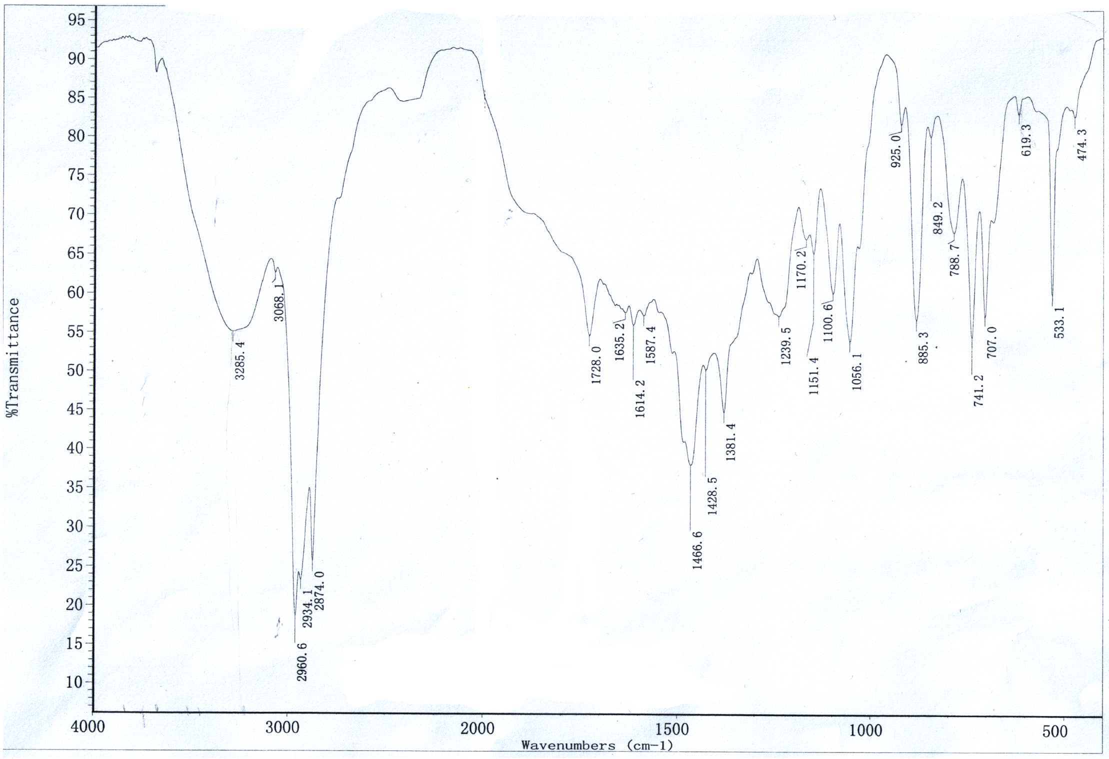

Figure S16. IR spectrum of the product after the reaction of RdBS with F^-.

The Matlab program for calculating the plasmon resonance scattering intensity for one nanoparticle.

```
file = 'sample.jpg';
image = imread(file);
imgGray = mat2gray(image,[0 65536]);
bins = 100;

% findSpots is a function for searching
% the scattering spots in the image
spots = findSpots(imgGray);

% estimate the intensity of each spot
% by averaging the gray value of pixels in the spot
count = length(spots);
intensity = zeros(count,1);
for i = 1:count
    positions = spots{i};
    l = length(positions);
    pixelIntensity = zeros(l,1);
    for j = 1:l
        pixelIntensity(j) = imgGray(positions(j,1),positions(j,2));
    end
```

```
    intensity(i) = mean(pixelIntensity);
end

% use histogram to distribute the intensity of spots
[h,x] = hist(intensity,bins);
xlabel('intensity');
ylabel('Counts');
```

Reference

[1] L. Shi, C. Jing, W. Ma, D.-W. Li, J. E. Halls, F. Marken, Y.-T. Long, *Angew. Chem. Int. Ed.* **2013**, *52*, 6011-6014.
